# DNA Methylation Status in Cancer Disease: Modulations by Plant-Derived Natural Compounds and Dietary Interventions

**DOI:** 10.3390/biom9070289

**Published:** 2019-07-18

**Authors:** Karin Jasek, Peter Kubatka, Marek Samec, Alena Liskova, Karel Smejkal, Desanka Vybohova, Ondrej Bugos, Kristina Biskupska-Bodova, Tibor Bielik, Pavol Zubor, Jan Danko, Marian Adamkov, Taeg Kyu Kwon, Dietrich Büsselberg

**Affiliations:** 1 Division of Oncology, Biomedical Center Martin, Jessenius Faculty of Medicine, Comenius University in Bratislava, 036 01 Martin, Slovakia; 2 Department of Medical Biology, Jessenius Faculty of Medicine, Comenius University in Bratislava, 036 01 Martin, Slovakia; 3 Division of Oncology, Biomedical Center Martin, Jessenius Faculty of Medicine, Comenius University in Bratislava, 036 01 Martin, Slovakia; 4 Department of Obstetrics and Gynecology, Jessenius Faculty of Medicine, Comenius University in Bratislava, 036 01 Martin, Slovakia; 5 Department of Natural Drugs, Faculty of Pharmacy, University of Veterinary and Pharmaceutical Sciences, 612 42 Brno, Czech Republic; 6 Department of Anatomy, Jessenius Faculty of Medicine, Comenius University in Bratislava, 036 01 Martin, Slovakia; 7 Lambda Life JSC., 851 01 Bratislava, Slovakia; 8 Department of Histology and Embryology, Jessenius Faculty of Medicine, Comenius University in Bratislava, 036 01 Martin, Slovakia; 9 Department of Immunology, School of Medicine, Keimyung University, Dalseo-Gu, Daegu 426 01, Korea; 10 Weill Cornell Medicine in Qatar, Qatar Foundation-Education City, Doha 24144, Qatar

**Keywords:** cancer, DNA methylation patterns, epigenetic modulations, oncogenes, phytochemicals, plant-based foods, tumor suppressor genes

## Abstract

The modulation of the activity of DNA methyltransferases (DNMTs) represents a crucial epigenetic mechanism affecting gene expressions or DNA repair mechanisms in the cells. Aberrant modifications in the function of DNMTs are a fundamental event and part of the pathogenesis of human cancer. Phytochemicals, which are biosynthesized in plants in the form of secondary metabolites, represent an important source of biomolecules with pleiotropic effects and thus provide a wide range of possible clinical applications. It is well documented that phytochemicals demonstrate significant anticancer properties, and in this regard, rapid development within preclinical research is encouraging. Phytochemicals affect several epigenetic molecular mechanisms, including DNA methylation patterns such as the hypermethylation of tumor-suppressor genes and the global hypomethylation of oncogenes, that are specific cellular signs of cancer development and progression. This review will focus on the latest achievements in using plant-derived compounds and plant-based diets targeting epigenetic regulators and modulators of gene transcription in preclinical and clinical research in order to generate novel anticancer drugs as sensitizers for conventional therapy or compounds suitable for the chemoprevention clinical setting in at-risk individuals. In conclusion, indisputable anticancer activities of dietary phytochemicals linked with proper regulation of DNA methylation status have been described. However, precisely designed and well-controlled clinical studies are needed to confirm their beneficial epigenetic effects after long-term consumption in humans.

## 1. Introduction

DNA methylation is a common mechanism of gene silencing through transcriptional repression. Global DNA methylation is a characteristic event in the cell strongly linked with certain processes, such as X-chromosome inactivation, the repression of repeated elements, or genomic imprinting [1]. The initiation, promotion, and progression of carcinogenesis are regulated by both genetic and epigenetic events [2]. In the last decade, epigenetic modulations have been increasingly recognized as important targets of research in oncology. Epigenetic alterations, including DNA methylation status, chemical modifications of histones, or RNA mechanisms (e.g., microRNAs and long noncoding RNAs), are responsible for the control of gene expression by regulating gene transcription [3]. Most DNA methylation, catalyzed by DNA methyltransferases (DNMTs), represents essential physiological steps that ensure both cellular and tissue homeostasis, playing an irreplaceable role in numerous key functions of cells [4,5]. However, aberrant DNA methylation also contributes to pathologic processes in organisms, and DNA methylation is one of the frequent and key epigenetic mechanisms that regulates processes associated with neoplastic transformation in eukaryotic cells, i.e., changes in apoptosis, proliferation, cell cycles, differentiation, and invasiveness, so it is therefore important in the development of cancer [6]. Based on changes in these processes, cancer cells can gain substantial resistance to anticancer drugs or chemopreventive agents, and thus resistance to multiple drugs may develop in cancer patients, or it could initiate a neoplastic transformation in healthy individuals [7,8].

Importantly, the dysregulation of DNA methylation patterns is a frequently recognized cellular event during both the initiation and late stages of oncogenesis. To date, numerous research groups have established a crucial role of both hypermethylation of tumor suppressor genes and global hypomethylation of oncogenes in cancer development and progression. Both processes are clinically valid, diagnostic, and prognostic markers in cancer disease [9,10]. Furthermore, there is evidence suggesting that cancer-associated DNA hypomethylation could increase the instability of the genome via the activation of retrotransposons of otherwise silent genomic regions [11,12]. On the other hand, hypermethylation of the promoter regions of tumor-suppressor genes demonstrates its particular role in neoplastic transformation of the cell [13,14]. Hypermethylation of promoters may lead to the silencing of genes affecting important cellular signaling pathways. Some of them are considered to be hallmarks of cancer. These include aberrant cell cycle regulation, the induction of apoptosis, DNA repair machinery, or the control of other key cancer-related molecular networks. The overall impact of DNA methylation on the functioning of human organisms is multifactorial and is directed by certain endogenous and exogenous factors, such as age, gender, ethnic group, stage of cancer, environmental stress factors, lifestyle, drugs, and dietary factors. More and more research data have supported the hypothesis that specific environmental factors during critical developmental stages may influence the later risk of carcinogenesis in part via persistent reprogramming of DNA methylation patterns [15].

Bioactive phytochemicals, which are widely available and usually show minimal toxicity in an organism, have been intensively evaluated for their presumptive role as epigenetic modulators of the activity of genes and their consequent potential for use in cancer chemoprevention and/or therapy [3,16]. In this regard, “nutri-epigenetics” have gained considerable attention within oncology research, because in contrast to genetic changes, epigenetic alterations are reversible and thus potentially serve as an important tool to affect the initiation of carcinogenesis.

### 1.1. Source of Data

Research data published as English-language articles from the PubMed database were collected and analyzed. Relevant studies were retrieved through the use of “epigenetics or DNA methylation status or DNA hypomethylation or DNA hypermethylation or cancer or phytochemicals or plant natural substances or plant food or diet or cancer cells or animal model or clinical study” as either keywords or a medical subject heading (MeSH) term in searches of the PubMed bibliographic database. We focused primarily on recent scientific papers from the years 2014–2019.

### 1.2. Aim of the Study

We aimed to highlight the role of plant-derived compounds (phytochemicals) and plant-based diets in targeting changes in DNA methylation status associated with all stages of carcinogenesis, as well as its potential use in a chemoprevention clinical setting. The core of this review is the conclusion of data from preclinical and clinical research and an assessment of whether and how dietary phytochemicals modulate aberrant DNA methylation processes in cancer cells.

## 2. The Role of DNA Methylation Patterns as Potential Diagnostic/Prognostic Markers of Carcinogenesis

All processes of promoter hypermethylation/hypomethylation or global hypomethylation [17] interfere with so-called “methylation patterns”. These patterns, in contrast to normal tissue, change during tumorigenesis, making it a basis for whole-genome methylation studies. Using numerous molecular approaches, numerous methylation biomarkers have been discovered that have the potential to predict specific diagnoses, prognoses, therapeutic targets, as well as therapeutic responses [18].

The list of potential biomarkers, based on changes in DNA methylation, is long [19]. Several genes have been found aberrantly methylated in several types of tumors, and most of them could potentially serve as important prognostic or diagnostic biomarkers. However, besides for the *MGMT* gene, available studies are still experimental. Hypermethylation of one of the first epigenetic biomarkers that should be implemented in the clinic, the *GSTP* gene, may be used for the early diagnosis of prostate cancer. In combination with the detection of DNA methylation status in other tumor suppressor genes (TSGs) (i.e., *AOX1, APC, EDNRB*), the sensitivity of a prostate cancer diagnosis substantially increases [19,20]. Studies using DNA methylation as a prognostic biomarker have identified several aggressive tumors with an increased risk of rapid progression and/or relapse *(RASSF1* in breast, prostate, squamous cervical cancer; *CDH1* in squamous cervical cancer; *APC* in breast cancer; *CDH1* in squamous cervical cancer; *CDKN2A* in colorectal cancer) [19,21,22]. DNA methylation in certain genes could also represent an excellent biomarker for predicting the response to a treatment. The hypermethylation of the *MGMT* repair gene in glioblastomas causes the gene to silence, which reduces its repair activity and the removal of alkyl groups, thus predicting a response to the chemotherapeutic agent [19,23]. However, the altered methylation profile of many TSG promoter regions appears to have multiple functions as methylation markers: For example, the hypermethylation of the *BRCA1* repair gene should serve as (i) a diagnostic marker where a methylation signature is proposed to predict a sporadic risk of developing breast cancer in BRCA1 mutation carriers; (ii) a prognostic marker that stratifies and predicts death in advanced diagnosis; (iii) a therapeutic marker that is also suggested to determine the sensitivity of breast and ovarian cancer to a cross-linking agent [19,20]. *LINE-1* is often considered a surrogate pattern for global DNA methylation, and its overexpression caused by promoter hypomethylation leads to a less favorable prognosis in non-smallcell lung carcinoma and gastrointestinal stromal tumors [22,24].

In terms of progression and metastatic spreading, an aberrant methylation plays a key role in predicting recurrence and potential therapeutic strategies. There has been evidence in experimental studies that hypermethylation of the *Bin1* gene (also reduced expression identified in breast cancer tissues) is associated with poor prognosis in esophageal squamous cell carcinoma (ESCC) patients [25]. Reduced expression of the *Bin1* gene is associated with metastasis (MTS) in lymph nodes, resistance to apoptosis, and an epithelial-to-mesenchymal transition (EMT) of ESCC cells in vitro and in vivo. Using demethylation agents, *Bin1* gene expression was restored, thereby inhibiting EMT and MTS diffusion through the suppression of the PTEN/AKT signaling pathway, paving the way for use as a novel therapeutic tool [26]. Experimental analyses of breast cancer-specific biomarkers revealed the aberrant methylation status of seven genes (*BRCA1, DAPK1, MSH2, CDKN2A, PGR, PRKCDBP, RANKL*) that have an eminent effect on MTS spreading, making them perspective prognostic markers for breast cancer relapse risk [27].

The elimination of DNA repair genes by hypermethylation results in the accumulation of DNA damage and thus promotes cancer formation. The hypermethylation of DNA repair genes appears to be specific, compared to the aberrant methylation of other TSGs, in that it not only incites carcinogenesis, but also affects the susceptibility of tumors to chemotherapy based on DNA-damaging agents destroying tumor cells [28]. The epigenetic inactivation of DNA repair genes in tumors was demonstrated in several DNA repair pathways: nucleotide excision repair (NER), mismatch repair pathways (MMRs), and homologous recombination (HR) [29]. The methylation of DNA repair genes such as *MGMT* (mentioned above)*, BRCA1*, and *ERCC1* has raised the therapeutic responsiveness of various cancer types within experimental studies, giving potential therapeutic benefits to patients [30]. Moreover, the epigenetic shutdown of the MMR *MLH1* gene function is associated with increased tumor resistance to chemotherapeutic agents [28,31]. These experimental data are promising for the development of individual cancer treatment strategies depending on the methylation status of a patient’s DNA repair genes.

## 3. Regulatory Mechanisms Controlling DNA Methyltransferase Activity

As mentioned previously, methylation is catalyzed by enzymes from the DNMT family, including DNMT1, DNMT3a, and DNMT3b. DNMT3a and DNMT3b mediate de novo methylation, which is essential for genome regulation and development [32]. DNMT1 represents a dominant enzyme with a role in the maintenance of methylation patterns in newly synthesized DNA strands during replication of the genetic material in the cell [33]. Alterations in the enzymatic activity of methyltransferases are responsible for the transcriptional silencing of multiple genes [34]. Based on the crucial role of DNMT1 in the maintenance of the methylation status of DNA and subsequent regulation of gene expression, there are various control mechanisms associated with regulation and stability of the enzyme, including intrinsic and extrinsic factors that directly or indirectly affect DNMT bioactivity. Intrinsic factors controlling the activity of *DNMTs* represent a cascade at transcriptional as well as at post-transcriptional regulation. Transcriptional regulation of DNMTs consists of signaling pathway cascades, including RAS-AP-1, PI3/PKB, pRb/E2F, or P53/SP1 [35,36,37]. Their abnormal induction leads to an increased level of DNMTs and is associated with cancer development. Other mechanisms contributing to methyltransferase activity and stability are related to post-transcriptional auto-inhibitory regulation via the Replication Focus Targeting Sequence (RFTS) domain, which is crucial for the targeting of DNMT1 into replication loci, and the C*XX*C domain, which is responsible for binding to an unmethylated CpG site. Both domains are localized to a regulatory *N*-terminal region, and their interactions with the catalytic *C*-terminal region are important in the modulation of DNMT activity. Additionally, the *N*-terminal domain has a vital function as a mediator of interactions with DNA, proteins, or substrates [38,39]. Extrinsic factors participate in the modulation of stability and activity of DNMTs through interactions with the outer environment, representing various regulatory molecules that affect the regulation of enzyme activity at a post-translation level [40]. For instance, the regulation of DNMTs at a post-translation level is available allosterically via an interaction with molecular partners, such as PCNA, UHRF1, or USP7 [41,42]. Moreover, extrinsic factors, including nutritional elements and other bioactive molecules, have to be considered in the modulation of DNMT activity globally or in specific CpG sites.

### The Effects of Phytochemicals on DNA Methylation Patterns

Throughout history, plant-derived food was widely used in the prevention or treatment of various diseases [43,44,45]. The association between cancer and aberrant epigenetic modifications together with subsequent gene defects [46] began to be of interest in cancer research, especially at the end of the 20th century [46,47,48]. Considerable progress in the field of epigenetic has been connected to the development of various methods available to determine the methylation profiles of DNA [49]. Therefore, the study of epigenetic alterations, including the evaluation of the methylation status of genes, became one of the major areas of cancer research [50], with their potential reversibility making them promising targets for cancer preventive or therapeutic strategies [46]. Accordingly, evaluations of phytochemicals affecting cancer-related epigenetic modifications in the first years of the 21st century led to the demonstration of the protective efficacy of genistein [51], *trans*-resveratrol [52], or tea-derived phenolic epigallocatechin-3-gallate (EGCG) [53] against aberrations in DNA methylation in vitro or in vivo. However, the rapid progression concerning the beneficial effects of phytochemicals in epigenetic-associated changes implied in the carcinogenesis has gained increased interest in the last decade.

**Figure 1:**
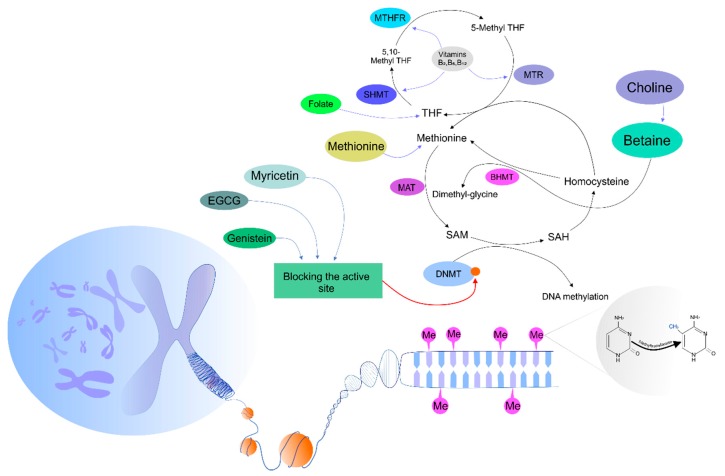
Mechanisms of phytochemicals affecting DNA methylation processes in the cell. DNMTs, DNA methyltransferases; SAM, S-adenosylmethionine; SAH, S-adenosyl-L-homocysteine; MAT, methionine adenosyltransferase; BHMT, betaine-homocysteine methyltransferase; THF, tetrahydrofolate; MTR, methionine synthase; MTHFR, methylenetetrahydrofolate reductase; SHMT, serine hydroxymethyltransferase.

Recent evidence has suggested a linkage between diet and methyltransferase activity [39]. There are three dominant options associated with nutrients and the methylation of DNA, including provision substrates important for methylation, alterations in enzymatic activity contributing to a one-carbon cycle, and the provision of cofactors modulating DNMT activity [54]. The modulation of the activity of methyltransferases by dietary intervention can be mediated via regulation of *S*-adenosylmethionine (SAM) synthesis. SAM represents the key donor of a methyl group and is a crucial substrate in methylation. Importantly, SAM is synthesized in a methionine cycle, in which numerous enzymes and essential nutrients present in food, including folate, methionine, choline, betaine, and vitamins, participate. These nutrients act as precursors in the methionine cycle and directly contribute to the generation of SAM. Therefore, a deficiency of the above-mentioned nutrients is associated with a dysregulation of the SAM level, leading to a lack of substrates for DNMT activity, thus dramatically altering DNA methylation and resulting in global hypomethylation [55]. Our knowledge about the role of nutrients acting as methyl donors comes from animal studies, in which the intake of a low-methyl donor diet was significantly associated with global hypomethylation and vice versa [56,57]. Accordingly, methyl donors from the diet are associated with the alteration of methylation status, but there are many uncertainties about doses and the duration of dietary exposure resulting in epigenetic changes of DNA [58]. As described previously, DNMTs require SAM as a cofactor. However, the activity of SAM may be modulated by dietary intervention, leading to the modulation of its concentration in the cell. Moreover, the activity and stability of methyltransferases is inhibited directly and competitively through interaction with natural compounds, including EGCG, a phenolic from green tea, genistein from soybean, or myricetin as the representative of dietary flavonoids [59,60,61]. A current study indicated that DNMT action could be disrupted by EGCG in human colon cancer cells via its binding to the active pocket of the enzyme [60]. Moreover, dietary isoflavone genistein was proposed as an antagonist of DNMT1 in MCF7 and MDA-MB-231 breast cancer cell lines [62]. Furthermore, myricetin (with a pyrogallol moiety structurally similar to a gallic moiety of EGCG) significantly inhibited human DNMT1 and prokaryotic SssI DNMT1 [39]. Importantly, cross-talk between nutrients and methylation status may lead to modifications of one-carbon metabolism through the regulation of enzymes participating in a methionine cycle. One-carbon metabolism represents a complex of biochemical processes with unique enzymes and corresponding coenzymes leading to the formation or utilization of methyl groups. The importance of the cycle lays in its ability to produce SAM from methionine and ATP [63,64]. One-carbon metabolism comprises methionine and folate cycles, which are tightly interconnected. Vitamins, such as B_2_, B_6_, and B_12_, play significant role during the enzymatic cascade of the one-carbon metabolic pathways. Interestingly, these cofactors regulate enzymes from the folate cycle that are associated with SAM bioavailability. Therefore, the above-mentioned cofactors of the folate cycle act as catalyzers of enzymatic activity, leading to a conversion of homocysteine (Hcy) into methionine, which is further converted via methionine adenosyl-transferase (MAT) into SAM. Subsequently, SAM is converted via glycine *N*-methyltransferase (GNMT) into *S*-adenosyl-homocysteine (SAH) [55]. In summary, numerous studies have focused on the cross-connection between diet and patterns of DNA methylation via one-carbon metabolism, donors of methyl groups from the diet, or the enzymatic activity of DNMTs. Importantly, all previously described regulatory mechanisms can operate synergistically in time, indicating the complexity and robustness of nutritional epigenomics processes. Figure 1 summarizes the mechanisms and effects of phytochemicals on DNA methylation status in the cell.

## 4. Preclinical Cancer Research

Epigenetic therapy is a novel clinical approach focused on the modulation of aberrant epigenetic changes, presumably at cancer-related genes, by chemicals [65,66]. Regarding the clinical concept of continually developing and enhancing the effectiveness of anticancer therapy and prevention, the use of phytochemicals with significant epigenetic properties has gained considerable interest over the last decades. Current findings from preclinical oncology research using cell lines and/or animal models have contributed to new knowledge in the field of plant-derived compounds as potential epigenetic agents with substantial effects on DNA methylation status to protect various cell types against neoplastic transformation.

### 4.1. In Vitro Studies

Several recent preclinical studies using cancer cell lines have demonstrated the modulation of DNA methylation patterns after treatment with phytochemicals. DNA methyltransferase (DNMT) activity was greatly reduced in MDA-MB-231 and MCF-7 cells after treatment with proanthocyanidins from grape seeds in combination with *trans*-resveratrol [67]. In addition, this combination of phytochemicals synergistically decreased cell viability and post-treatment cell proliferation in both cell lines and induced apoptosis in MDA-MB-231 cells with a noticeable increase in the ratio of Bax/Bcl-2 expression. In another study using the HT29 cell line, indicaxanthin, a plant pigment present in beets, showed antiproliferative activity and induced demethylation in the promoters of certain methylation-silenced tumor-suppressor genes (i.e., *p16INK4a*, *GATA4*, and *ESR1*) that are involved in colorectal carcinogenesis [68]. Indicaxanthin also upregulated the expression of genes involved in DNA demethylation: Moreover, in silico molecular modeling revealed stable binding of this phyto-substance at the DNMT1 catalytic site. Jiang et al. [69] documented that glabridin, a phytochemical from the root of *Glycyrrhiza glabra*, enhanced the expression of miR-148a through DNA demethylation in MDA-MB-231 and Hs-578T breast cancer cell lines. Consequently, miR-148a blocked the expression/activation of SMAD2 (via targeting the SMAD2-3′-UTR) and thus restored epithelial characteristics, adhesive abilities, and cancer stem cell-like properties. Wong et al. [70] aimed to evaluate the genome-wide effects of sulforaphane (SFN) and 3,3′-diindolylmethane (DIM) on DNMT1, DNMT3A, and DNMT3B activity and promoter methylation status in normal prostate epithelial cells and LnCAP and PC3 prostate cancer cell lines. For all three prostate cell lines, the authors revealed widespread changes in promoter methylation patterns in response to SFN or DIM interventions. Importantly, SFN and DIM reversed many of the cancer-associated methylation alterations, including aberrantly methylated genes that are dysregulated or are highly involved in cancer progression, such as C–C chemokine receptor type 4 (CCR4), transforming growth factor-β1 receptor type I (TGFBR1), cysteine-rich angiogenic inducer 61 (CYR61), and C-X-C chemokine receptor type 4 (CXCR4). Suberoylanilide hydroxamic acid (SAHA), a histone deacetylase (HDAC) inhibitor used in the treatment of cutaneous T-cell lymphoma, was administered in combination with EGCG, a DNA methyltransferase (DNMT) inhibitor, against MDA-MB-231 and MDA-MB-157 (triple-negative) breast cancer cell lines. The combined therapy decreased the activity of DNMTs. In this regard, the combination of compounds positively affected the expression of oncogenic miR-221/222, tumor suppressors p27 and PTEN, and ERα. An increased ratio in E-cadherin/N-cadherin gene expression indicated a more epithelial phenotype. These data pointed to the reduced migration of triple-negative breast cancer cells [71]. DNMT1, as the major enzyme responsible for the maintenance of the DNA methylation pattern, can potentially be used as an anticancer target. In this regard, solamargine, a poisonous chemical compound occurring in plants of the Solanaceae family, has been described as regulating EP4 downstream c-Jun via ERK1/2-mediated attenuation of DNMT1 activity in human nonsmall-cell lung cancer in vitro [72].

There are some reports that have demonstrated an increased sensitivity of cancer to conventional therapy after the administration of phytochemicals. Szarc Vel Szic et al. [73] showed that withaferin A (WA) downregulated HER2/PR/ESR-dependent gene expression interactions and thus repressed aggressive triple-negative MDA-MB-231 breast cancer cells with a specific DNA hypomethylation profile. In contrast to the DNA demethylating agent 5-aza-2′-deoxycytidine, WA treatment of MDA-MB-231 cells affected an epigenetic signaling network through gene-specific DNA hypermethylation of tumor oncogenes. This included a urokinase-type plasminogen activator, ADAM metallopeptidase domain 8, tumor necrosis factor (ligand) superfamily member 12, and genes related to mitochondrial metabolism (malic enzyme 3, ME3) and enzymes of cell detoxification, such as glutathione *S*-transferase mu 1. Based on this, WA may represent an attractive natural compound for the treatment of triple-negative breast cancer (TNBC), with the potential to improve therapeutic sensitivity to conventional drugs via epigenetic mechanisms. Another study revealed that physiologic concentrations of dietary phytochemicals, such as curcumin, DIM, EGCG, genistein, or indole-3-carbinol (I3C), via changes in DNA methylation, altered the gene expression of cadherin-11, p21Cip1, urokinase-type plasminogen activator, and interleukin-6 and consequently reduced the growth and apoptosis of MDA-MB-231 cancer cells [74]. The authors concluded that future research focused on the modulation of specific gene expression and cellular pathways by dietary agents is necessary and might offer a clinical tool to enhance the sensitivity of cancer cells to anticancer drugs. Li et al. [75] aimed to evaluate in vitro and in vivo epigenetic effects of genistein on *ERα* reactivation in MDA-MB-231 breast cancer cells. Moreover, the authors evaluated the expression of DNMT1 and HDAC1 as the most important epigenetic enzymes accompanied with the expression of changes of *ERα*. Changes in enzymatic activities of DNMTs in vitro and in vivo after genistein treatment pointed to the fact that DNA methylation may modify *ERα* expression via the regulation of DNMT-involved transcription. These data suggest that DNA methylation plays a role in genistein-induced *ERα* reactivation in ER-negative BC cells and thus induces the sensitivity of BC cells to endocrine therapy, e.g., with tamoxifen [75]. All above-mentioned results are summarized in Table 1.

### 4.2. In Vivo Studies

The importance of naturally occurring plant-derived compounds in the modulation of methylation patterns has been supported by numerous studies using animal models. Dietary phenolics such as curcumin, which is a major compound of turmeric, are associated with beneficial impacts on health via antioxidant and anti-inflammatory features [76]. There have been several studies focusing on the role of curcumin in epigenetic regulations, including methylation. Du et al. [77] evaluated the effect of curcumin (peritoneal administration as solution in dose 100 mg/kg) on the reactivation of the tumor-suppressor gene *RASSF1* in female athymic nu/nu mice in experimental mammary carcinogenesis using inoculated MCF-7 cells. Curcumin decreased tumor size, which correlated with specific CpG site hypomethylation of the *RASSF1* promoter [77]. Similarly, curcumin significantly decreased the methylation status of the RARβ promoter region, leading to gene reactivation in female BALB/c nude mice in a lung cancer experimental model [78]. Moreover, in an experimental model of acute myeloid leukemia in female athymic nu/nu mice, a decreased expression of *DNMT1* was found after treatment with curcumin [79]. Furthermore, an effect of curcumin associated with DNA methylation was detected in prostate tumorigenesis in TRAMP mice. Curcumin intervention decreased the methylation status of the *Nrf2* promoter region [80]. Genistein possesses numerous biological activities, including antioxidant, anti-inflammatory, and anticipative features. Importantly, there has been evidence suggesting a role of genistein in the modulation of DNA methylation patterns [81]. Genistein was associated with the reactivation of estrogen receptor-α (ERα) as a consequence of lowered expression of *DNMT1* in a breast cancer experimental model in virgin female immunodeficiency nu/nu mice [75]. Genistein as an epigenetic modifier also decreased tumor growth and methylation status in the *CDH5* promoter region via the downregulation of *DNMT3* in a neuroblastoma model, thus acting as an inhibitor of DNA methyltransferase activity in vivo [82]. *Trans*-resveratrol is a nutritional compound associated with numerous therapeutic effects, including the regulation of cancer initiation and progression [83]. This phytochemical demonstrated significant antineoplastic properties in experimental mammary carcinogenesis via the downregulation of *DNMT3* expression in ACI rats [84]. Kaempferol is a flavonoid occurring in many vegetables and fruits that demonstrates health benefits for humans [85]. The administration of kaempferol led to the downregulation of *DNMT3* and the demethylation of more than 100 DNA methylation positions associated with genes in an experimental model of bladder carcinogenesis in vivo [86]. The dietary chalcone isoliquiritigenin, which is found mainly in licorice root, significantly inhibited breast cancer progression via the demethylation of the *WIF1* promoter region, which correlated with the inhibition of *DNMT1* in a xenograft mouse model [87]. A lot of preclinical in vivo studies have focused on the mixture of phytochemicals in plants and their impact on the regulation of methylation patterns in various malignancies. Our group demonstrated that the mixture of phytochemicals present in *Thymus vulgaris* L. is significantly linked with tumor-preventive effects in an animal model of chemically induced breast carcinoma. Thyme haulm was administrated in a diet, and an evaluation of the DNA methylation status of five promoters of tumor-suppressor genes (*ATM, PITX2, RASSF1, PTEN*, and *TIMP3*) showed significant decreases in methylation patterns in vivo after intervention with thyme [88]. Furthermore, our previous experiment using the same animal model focused on the epigenetic modulation of clove buds and showed a demethylation effect on the promoter of the *RASSF1* tumor suppressor in mammary tumors in vivo [89]. Black raspberries (BRBs) represent a mixture of vitamins, minerals, fiber, anthocyanin, and other compounds, showing antioxidant and anti-inflammatory properties in cancer [90]. Dietary administration of BRBs led to the demethylation of promoters of tumor-suppressor genes, including *WIF1*, *SOX17*, and *GKI*, in precancerous colon cancer in vivo [91]. Moreover, BRBs showed a demethylation effect via the reduction of the methylation status of the *Sfr4* promoter region in a rat esophageal squamous cell papilloma model. This gene plays a role as an antagonist of WNT signaling pathways involved in the development of human squamous cell carcinoma [92]. An overview of phytochemicals targeting DNA methylation in cancer animal studies is shown in Table 2.

## 5. Dietary Intervention and DNA Methylation Patterns in Cancer Clinical Research

As stated above, human malignancies are characterized by aberrant changes in DNA methylation, mainly hyper- or hypomethylation. The tumor cell genome may be globally hypomethylated, or the hypermethylation can occur in numerous CpG islands associated with gene promoters [17,93]. Several clinical trials have investigated the role of phytochemicals or other micronutrients as anticancer agents, with their efficacy mediated via the modulation of changes in DNA methylation. Due to the fact that DNA methylation is catalyzed via DNMTs [93], their inhibitors are important anticancer agents. Synthetic DNMT inhibitors, such as 5-aza-2′-deoxycytidine (5Aza-C), are narrow in their activity and may cause adverse toxic events. On the contrary, dietary phytochemicals show fewer side effects, are widely available, and act as direct or indirect DNMT inhibitors [94]. Majid et al. [95] evaluated the efficacy of genistein and the DNA methyltransferase inhibitor 5Aza-C on the expression of *BTG3* through analysis of promoter methylation and chromatin modifications in prostate cancer cell lines and tissue samples obtained from patients of radical prostatectomy. Importantly, the *BTG3* promoter region was hypermethylated in both cell lines and tumor samples when compared to the control. Nevertheless, the administration of genistein (50 µM) led to the demethylation of the hypermethylated *BT3G* promoter and thus reactivated expression of the gene, although to a lesser extent than of 5Aza-C [95]. Furthermore, premenopausal women with no history of atypia, in situ, or invasive breast cancer were included in the study, which dealt with the effects of soy isoflavone and circulating genistein on estrogenic markers and methylation levels of five cancer-related genes. Subsequently, the administration of isoflavones for one menstrual cycle in healthy premenopausal women led to significant alterations in the methylation levels of *RARβ2* and *CCND2* in the breast tissue. Importantly, these changes were dependent on the levels of circulating genistein due to the close link between the decrease in *RARβ2* methylation and a post-treatment genistein level less than 600 ng/mL and the decrease in *CCND2* methylation and post-treatment genistein less than 200 ng/mL. Paradoxically, post-treatment with a greater level of genistein led to an increase in the methylation of both genes. Despite statistically significant alterations in the methylation status, the biological importance of these changes is uncertain, but at least different mechanisms of action for high and low doses of isoflavones is suggested [96]. Moreover, a dose-dependent effect of *trans*-resveratrol on a hypermethylated promoter was demonstrated in a study conducted on subjects with an increased risk of breast cancer. An increase in the levels of serum *trans*-resveratrol was significantly associated with a decrease in methylation of *RASSF-1α*, while these changes in methylation were related to a decrease in systematic and breast-specific prostaglandin (PGE2) expression [97].

Folates are water-soluble vitamins that function as methyl donors in a one-carbon metabolism cycle [98] and thus modulate the stability of DNA. Consequently, the regulation of DNA biosynthesis, repair, and methylation is highly dependent upon the intake of folate in the diet [99,100], while aberrant changes in folate metabolism may contribute to the development of cancer [101]. Actually, *RARB*, *BRCA1*, and *RASSF1A* are genes frequently methylated in breast cancer. A study analyzing an association between folate and other one-carbon-related nutrients (including vitamins B_2_, B_6_, and B_12_) and methionine and promoter hypermethylation and the expression of these genes was conducted on Iranian women with primary breast cancer. The results revealed that the dietary intake of folate and cobalamin was inversely associated with the methylation status of *RARB* and *BRCA1*. Therefore, a low intake of folate and cobalamin correlated with the age-dependent tendency of promoter regions of these genes to be hypermethylated in tumors. However, a high dietary intake of riboflavin and pyridoxine was found to be a determinant of the *RARB* methylated promoter when compared to consumers of low amounts and thus contribute to the development of breast tumors. The results of the study thus suggest the possibility of age-dependent selectivity of dietary components on hypermethylation status [102]. Colacino et al. [103] evaluated the effects of the pretreatment dietary intake of micronutrients involved in one-carbon metabolism, antioxidants, and food groups abundant in these nutrients on DNA methylation in newly diagnosed head and neck patients. The analysis considered the effect of diet on the methylation of individual CpG regions of selected genes (*RARB, C4B, GML, PTHR1, MLF1, THPO, CHGA, XIST, LCN2, SRC, SFN, PTHLH, LMO2, SIN3B, TNFSF8, HGF, HOXA11*) and revealed that subjects reporting in the highest quartile of folate, vitamin B_12_, and vitamin A intake showed decrease in promoter methylation of tumor suppressors when compared to subjects in the lowest quartile, as was also demonstrated in subjects reporting a high intake of cruciferous vegetables, which is a rich source of folate [103]. Similarly, low dietary folate led to the methylation of *p16^INK4A^*, the methylation silencing being related to functional polymorphism in methylene tetrahydrofolate reductase, a folate metabolism enzyme. The results suggest that there may be a connection between the low intake of folate, a well-known risk factor for head and neck squamous cell carcinoma (HNSCC), and the inactivation of *p16^INK4A^* in HNSCC [104]. Interestingly, there have been other studies supporting the relation between folate intake and a decrease in the methylation of tumor-suppressive genes. It is supposed that DNA methylation in colorectal cancer is also associated with physiological and environmental factors, including the involvement of one-carbon metabolism. Therefore, the level of promoter methylation was evaluated in several genes in colorectal cancer samples and healthy adjacent tissues of subjects confirmed for colorectal cancer. The results showed a link between plasma folate levels below normal and a higher level of methylation of the h*MLH1* promoter region [101]. On the contrary, the contribution of dietary factors involved in one-carbon metabolism with aberrant DNA methylation was evaluated in women diagnosed with primary incident breast cancer. However, no association between the dietary intake of folate, vitamins B_2_, B_6_, and B_12_, and methionine and promoter methylation of E-cadherin, *p16*, and *RAR-β2* genes was found [105].

Apart from changes in DNA methylation in gene promoters, global DNA hypomethylation is also closely linked to carcinogenesis [93,106]. Folate and vitamin B_12_ deficiency and global DNA hypomethylation was observed in subjects with squamous cell lung cancer when comparing samples of tumor and adjacent tissue, which appeared to be normal from the same patients [107]. Due to the fact that low folate status increases the risk of colorectal cancer, the effects of folic acid supplementation on DNA biomarkers were evaluated in colonocytes from adenomatous polyps and tissue adjacent to the former polyp site. Overall, folic acid supplementation decreased global DNA hypomethylation, increased tissue folate, and decreased the uracil misincorporation ratio when compared to a placebo, thus improving the DNA biomarkers of cancer risk [108]. Additionally, a proper dietary pattern is surely included in the notion of a lifestyle and may also influence DNA methylation patterns, as was demonstrated by Delgado-Cruzata et al. [106], who analyzed the impact of lifestyle preferences on the epigenetic indicators of blood DNA methylation level, including global DNA methylation by the LUMA assay targeting CCGC sequences throughout the genome, DNA methylation of the repetitive element LINE-1, and tandem repeat Sat2. After all, it was observed that dietary modification and weight loss generally attributed to a healthier lifestyle affected changes in global DNA methylation in Hispanic, Afro-American, and Afro-Caribbean breast cancer survivors. Specifically, DNA methylation of LINE-1 was increased when compared to baseline after 6 and 12 months. Moreover, positive associations between alterations in the diet pattern, including changes in vegetable, protein, and total caloric intake, LUMA DNA methylation, and the intake of fruit, leading to positive changes in DNA methylation of LINE-1, were also observed [106]. As the LINE-1 was hypomethylated in tumor tissues of colon cancer, the epigenetic changes in the surrounding tissue should also be investigated. Therefore, an analysis of the impact of serum vitamin D and obesity on global hypomethylation was performed in the visceral adipose tissue from patients with and without colorectal cancer. Interestingly, the DNA methylation of LINE-1 was positively associated with the level of vitamin D and negatively with BMI, and the insulin resistance index with serum vitamin D was the main variable of LINE-1 variance [109].

The dietary intake of genistein, *trans*-resveratrol, folate, and various vitamins contributes to changes in DNA methylation. The results of clinical trials are summarized in Table 3. Significantly, several clinical studies have shown that proper dietary intake of these nutrients led to the remodeling of aberrant changes in DNA methylation in cancer patients, healthy subjects, and subjects with increased risk of developing cancer. However, results from the overall analysis of available clinical studies are not as clear, as there are unexpected but surely interesting findings suggesting the relation between micronutrient efficacy and the dose or age of study participants. Evidence from clinical trials has shown the positive effects of dietary intervention on DNA methylation patterns. Nonetheless, we highly support clinical research being further extended so that results that are more concrete can be obtained and progress in anticancer research can be made.

## 6. Conclusions and Perspectives

To date, it has been well described that epigenetic alterations dynamically contribute to cancer pathogenesis. The preclinical and clinical evidence has demonstrated that plant-based molecules may decrease the risk of the development of certain types of neoplasia. Importantly, clinical studies have clearly indicated that the regular dietary intake of phytochemicals can lead to the remodeling of aberrant changes in DNA methylation in cancer patients and high-risk or healthy subjects. Specifically, these natural molecules can potentially show benefits due to their reverting activities in the epigenetic modulations of oncogenes and tumor-suppressor genes, as well as in their ability to modify and reinstate the expression of cellular protein levels. In addition, it is proposed that phytochemicals enhance the anticancer activity of conventional drugs used in clinical practice. Demethylating agents (such as inhibitors of DNMTs) modify gene expression by reversing the aberrant epigenetic alterations that are characteristic features of the running carcinogenesis. Numerous laboratories have documented that natural compounds or whole-plant foods sensitize tumor cells through different epigenetic targets, including oncogenes and tumor suppressor genes, as well as DNMTs and other epigenetic mechanisms, such as chemical modifications of histone molecules and changes in the expression of miRNA or long noncoding RNA. In addition, the combination of isolated phytochemicals or plant foods with chemotherapeutic agents could provide an additive or synergistic anticancer effect, enhancing the final therapeutic outcome. An understanding of the complexity of epigenetic targets and epigenetic molecular signaling pathways to elicit the efficacy of plant natural compounds and their mechanisms that are involved in tumorigenesis, especially in cancers with a poor prognosis (i.e., highly aggressive and invasive lesions that demonstrate resistance to conventional therapies), should be beneficial for clinical oncologists. The role of the epigenetic diet and its impact on the cancer epigenome is highly clinically relevant. To this end, recently published clinical data provide an excellent example on the functional link between the metabolism of homocysteine as a well-known epigenetic modulator and the etiology of highly aggressive premenopausal triple-negative breast cancer (TNBC): As a matter of fact, specifically a mild increase in total homocysteine, usually resulting from dietary habits, but not hyperhomocysteinemia as a genetic deficit, has been attributed to the TNBC pathology [110]. Consequently, the mild epigenetic modulation of tumor suppressors resulting in cancer reprogramming and persistent promotion pressure toward metastatic disease have been hypothesized. Contextually, an identification of the transformation-specific multiomic signature is essential for predictive diagnostics and targeted treatments [110,111,112,113]. In consensus, an advanced approach through predictive, preventive, and personalized medicine is considered to be the medicine of the future in overall cancer management [114].

It is necessary to establish more comprehensive studies focused on the epigenetics diet that will be able to reveal important translational information for cancer prevention and combinational therapy. Follow-up studies are essential to evaluate the safety profile of doses used, the route of administration, tissue distribution, and the bioavailability of natural plant compounds administered alone or in combination with other anticancer drugs with the aim to attain the best clinical outcomes. In addition, future research should be directed toward different clinical settings in humans, using a personalized approach to establish the potential of phytochemicals in individuals. Finally, a deeper understanding of the global patterns of epigenetic changes caused by natural plant compounds in cancer cells will lead to designing improved clinical strategies to prevent and cure cancer disease.

## Figures and Tables

**Table 1:** DNA methylation patterns in cancer cell lines after treatment with plant natural compounds. SAHA: Suberoylanilide hydroxamic acid.

Phytochemicals	Cancer Type	Cell Line	Effects on DNA Methylation and/or Gene Expression	Reference
Grape seed proanthocyanidins + *trans*-resveratrol	Breast	MDA-MB-231, MCF-7	Decreased DNMT activity	[67]
Indicaxanthin	Colon	HT29	Gene promoter demethylation of *p16INK4a*, *GATA4*, *ESR1*, hypermethylation of *SFRP1* and *HPP1*	[68]
Glabridin	Breast	MDA-MB-231, Hs-578T	Gene promoter demethylation of miR-148a	[69]
Sulforaphane and 3,3′-diindolylmethane	Prostate	LnCAP, PC3	Gene promoter methylation changes in *CCR4*, *TGFBR1*, *CYR61*, and *CXCR4*	[70]
Epigallocatechin-3-gallate + SAHA	Breast	MDA-MB-231, MDA-MB-157	Decreased activity of *DNMTs*, decreased gene expressions of miR-221/222, *p27*, *PTEN*, ERα	[71]
Solamargine	Lung	H1299, A549	Inhibited protein expression of *DNMT1* via activation of *ERK1*/2	[72]
Withaferin A	Breast	MDA-MB-231	Hypermethylation of oncogenes *PLAU*, *ADAM8*, *TNSF12*, *GSTM1*, and *ME3*	[73]
Curcumin, 3,3′-diindolylmethane, epigallocatechin gallate, genistein, indole-3-carbinol	Breast	MDA-MB-231	Changes in DNA methylation/gene expression of *CDH11*, *p21Cip1*, *PLAU*, and *IL-6*	[74]
Genistein	Breast	MDA-MB-231	Increase of ERα expression via the regulation of DNMT1-involved transcription	[75]

**Table 2:** Phytochemicals targeting DNA methylation in cancer animal model studies.

Phytochemicals (Isolated/Mixture)	Type of Cancer	Animal Model	Effects on DNA Methylation and/or Gene Expression	Reference
Curcumin	Breast	Female athymic nu/nu mice	Decrease in promoter methylation status of *RASSF1*	[76]
	Lung	BALB/c nude mice	Decrease in promoter methylation status of *RARβ*	[78]
	Acute myeloid leukemia	Female athymic nu/nu mice	Decrease in *DNMT1* expression	[79]
	Prostate	TRAMP mice	Decrease in promoter methylation status of *Nrf2*	[80]
Genistein	Breast	Female immunodeficiency nu/nu mice	Decrease in *DNMT1* expression	[75]
	Neuroblastoma	BALA/c nude mice	Demethylation of *CDH5* promoter, decrease in *DNMT3* activity	[82]
*Trans*-resveratrol	Breast	ACI rats	Decrease in *DNMT3* expression	[84]
Kaempferol	Bladder	BALB/c nude mice	Decrease in *DNMT3* expression	[86]
Isoliquiritigenin	Breast	MMTV-PyMT mice	Demethylation of *WIF1* promoter, decrease in DNMT1 activity	[87]
*Thymus vulgaris* L.	Breast	Rat model	Decrease in methylation status of *ATM, RASSF1*, *PTEN*, and *TIMP3* promoters	[88]
Clove buds	Breast	Rat model	Decrease in methylation status of *RASSF1 promoter*	[89]
Black raspberries	Colon	IL-10 KO mice	Decrease in methylation status of *WIF1*, *SOX17*, and *GKI* promoters	[91]
	Esophageal	Rat model	Decrease in methylation status of *Sfr4* promoter	[92]

**Table 3:** Clinical trials describing the effects of phytochemicals on DNA methylation patterns in cancer disease.

Dietary Intervention	Dosage	Study Design	Subjects Characteristics (n)	Dietary Intake-Based Methylation Changes	Reference
Genistein 5Aza-C	Different doses	-	Prostate cancer patients	Genistein and 5-Aza-C treatment: ↓ *BTG3* promoter methylation	[95]
Isoflavones—circulating genistein	40 mg/d or 140 mg/d	Prospective, double-blind, randomized trial	Healthy premenopausal women (*n* = 34)	Lower level of genistein: ↓ *RARβ2* and *CCND2* methylation Higher level of genistein: ↑ *RARβ2* and *CCND2* methylation	[96]
*Trans*-resveratrol	10 mg/d or 100 mg/d or placebo	Prospective, double-blind, and placebo-controlled study	Women with increased risk for breast cancer (*n* = 39)	↑ Trans-resveratrol: ↓ *RASSF-1α* methylation	[97]
Folate, B_2_, B_6_, B_12_, methionine	Dietary intake estimated via questionnaire	Prospective case cohort study	Primary breast cancer patients (*n* = 139)	↑ Riboflavin and pyridoxine: ↑ *RARB* promoter methylation; folate and cobalamin: Age-dependent correlation with the methylation status of *RARB* and *BRCA1*	[102]
Folate, vitamin B_12_, Vitamin A, cruciferous vegetables	Dietary intake estimated via questionnaire	Cross-sectional study	First primary head and neck cancer patients (*n* = 49)	↑ Folate, vitamin B_12,_ and vitamin A: ↓ Tumor suppressors methylation	[103]
Folate	Dietary intake estimated via questionnaire	Population-based study	Head and neck squamous cell cancer patients (*n* = 169)	↓ Folate: ↑ *p16^INK4A^* methylation	[104]
Folate	-	-	Colorectal cancer patients (approx. *n* = 40)	↓ Folate: ↑ h*MLH1* promoter methylation	[101]
Folate, B_2_, B_6_, B_12_, methionine	Dietary intake estimated via questionnaire	Population-based case-control study	Primary breast cancer patients (*n* = 1170)	No association found	[105]
Folate, vitamin B_12_ deficiency	-	-	Squamous cell lung cancer patients (*n* = 12)	↓ Folate, vitamin B_12_: Global DNA hypomethylation	[107]
Folic acid supplementation	600 μg/d or placebo	Randomized controlled trial	Patients with adenomatous polyps (*n* = 20)	↑ Folic acid supplementation: ↓ Global DNA hypomethylation	[108]
Dietary modifications (vegetable, proteins, changes in caloric intake) and weight loss		Randomized, crossover, pilot study	Hispanic, African-American, and Afro-Caribbean overweight and sedentary breast cancer survivors (*n* = 24)	↑ LINE-1	[106]
25-hydroxyvitamin D	-	-	Colorectal cancer patients (*n* = 55) and control subjects (*n* = 35)	↑ 25-hydroxyvitamin D: ↑ LINE-1	[109]

Explanatory notes: ↑ increase; ↓ decrease. Abbreviations: 5Aza-C–5-aza-2′-deoxycytidine; *BRCA1*, breast cancer-1; *BTG3*, B-cell translocation gene 3; h*MLH1*, MutL Homolog 1; *LINE1*, long interspersed nuclear element-1; *p16^INK4^*, *cyclin-dependent kinase inhibitor 2; RARB*, retinoic acid receptor-beta; *RARβ2*, retinoic acid receptor-beta 2; *RASSF-1α*, Ras association domain family-1 isoform.
